# A note of thanks to referees in 2010

**Published:** 2010

**Dementia & Neuropsychologia** finishes the fourth year of publication and we would like to thank our referees for sharing their expertise with us. The high quality of constructive criticisms and dedication of all our reviewers are highly appreciated by both the editors and the authors.

**Table:** 

Alexandra Reis, Lisboa, Portugal Ana Paula de Oliveira Santana, Campinas, SP Analuiza Camozzato de Pádua, Porto Alegre, RS Andrea Corsonello, Cosenza, Italy Andreia Camaz Deslandes, Rio de Janeiro, RJ Anja Lowit, Glasgow, United Kingdom Beatriz Baldivia, São Paulo, SP Benito Pereira Damasceno, Campinas, SP Bernadette Ska, Montréal, Canada Breno Satler de Oliveira Diniz, São Paulo, SP Carla Cristina Guariglia, São Paulo, SP Carolyn Zhu, Nanjing, China Cassio Machado de Campos Bottino, São Paulo, SP Ceres Eloah de Lucena Ferretti, São Paulo, SP Cristiana Borges Pereira, São Paulo, SP Dagoberto Callegaro, São Paulo, SP Daniel Deheinzelin, São Paulo, SP Lidia Mayumi Nagae, Philadelphia, USA Egberto Reis Barbosa, São Paulo, SP Eliane Correa Miotto, São Paulo Eliasz Engelhardt, Rio de Janeiro, RJ Érica de Araújo Brandão Couto, Belo Horizonte, MG Fábio Henrique de Gobbi Porto, São Paulo, SP Fernanda Alves da Cruz Gouveia Paulino, São Paulo, SP Fernando E. Taragano, Buenos Aires, Argentina Francisco de Assis Carvalho do Vale, Ribeirão Preto, SP Gabriel Rodriguez de Freitas, Rio de Janeiro, RJ Gabriela Coelho Pereira De Luccia, São Paulo, SP Gerson Chadi, São Paulo, SP Giuliano Emerenciano Ginani, São Paulo, SP Hélio Afonso Ghizoni Teive, Curitiba, PR Henrique Cerqueira Guimarães, Belo Horizonte, MG Isabel Albuquerque M. de Carvalho, São Paulo, SP Ivan Hideyo Okamoto, São Paulo, SP Jacqueline Abrisqueta-Gomez, São Paulo SP Jerson Laks, Rio de Janeiro, RJ João Carlos Barbosa Machado, Belo Horizonte, MG João Eliezer Ferri-de-Barros, São José dos Campos, SP José Roberto Wajman, São Paulo, SP Julia Jaekel, Berlin, Germany Juliana Nery de Souza-Talarico, São Paulo, SP Kara L. Bopp, Clinton, NY Karla Rodrigues Cavalcante, Taubaté, SP Leandro Tavares Lucato, São Paulo, SP	Lendro Malloy Diniz, Belo Horizonte, MG Leonardo Ferreira Caixeta, Goiânia, GO Leticia Lessa Mansur, São Paulo, SP Luis Sidônio Teixeira da Silva, Londrina, PR Luiz Augusto Franco de Andrade, São Paulo, SP Luiz Henrique Martins Castro, São Paulo, SP Maira Tonidandel Barbosa, Belo Horizonte, MG Marcia Lorena Fagundes Chaves, Porto Alegre, RS Marcia Maria Pires Camargo Novelli, Santos, SP Marcia Radanovic, São Paulo, SP Marco Antônio Rosa Machado, Campinas, SP Maria Isabel d’Ávila Freitas, São Paulo, SP Maria Joaquina Marques-Dias, São Paulo, SP Maria Lúcia Gurgel da Costa, Pernanbuco, PE Maria Teresa Carthery-Goulart, São Paulo, SP Marina Ceres Silva Pena, Ribeirão Preto, SP Martin Andreas Metzger, São Paulo, SP Mauro Muszkat, São Paulo, Sp Eliane Mayumi Kato-Narita, São Paulo, SP Mirna Lie Hosogi Senaha, São Paulo, SP Mirna Wetters Portuguez, Porto Alegre, RS Mônica SanchesYassuda, São Paulo, SP Nelson Ferreira Fortes, São Paulo, SP Maria Paula Foss, Ribeirão Preto, SP Paulo Caramelli, Belo Horizonte, MG Paulo Eduardo Mestrinelli Carrilho, Cascavel, PR Paulo Mattos, Rio de Janeiro, SP Paulo Renato Canineu, São Paulo, SP Renata Areza-Fegyveres, São Paulo, SP Renata Eloah de Lucena Ferretti, São Paulo, SP Renato Anghinah, São Paulo, SP Ricardo de Oliveira Souza, Rio de Janeiro, SP Roberto Alves Lourenço, Rio de Janeiro, RJ Robin Lea West, Gainesville, FL Rodrigo Rizek Schultz, São Paulo, SP Rogerio Beato, Belo Horizonte, MG Sara Joyce Shammah Lagnado, São Paulo, SP Sonia Maria Dozzi Brucki, São Paulo, SP Tales Alexandre Aversi-Ferreira, Toyama, Papan Valeria Santoro Bahia, São Paulo, SP Valeska Marinho Rodrigues, Rio de Janeiro, RJ Viviane Hiroki Flumignan Zétola, Curitiba, PR

